# Medical and Philosophical Intelligence

**Published:** 1815-05

**Authors:** 


### MEDICAL AND PHILOSOPHICAL INTELLIGENCE.

*Officers and Council of the Medical and Chirurgical Society of London: elected March 1st,* 1815.—*President,* Henry Cline, Esq. F.R.S.—C. R. Aikin, Esq.—Thomas Bateman, M.D. F.L.S. Librarian.—Charles Bell, Esq. F.R.S. Ed.—George Birkbeck, M.D.—Sir Gilbert Blane, Bart. M.D. F.R.S.—John Bright, M.D.—Benjamin C. Brodie, Esq. F.R.S.—Astley Cooper, Esq. F.R.S. Treasurer.—Alexander Henderson, M.D.—William Lawrence, Esq. F.R.S. Secretary.—Sir James Macgrigor, M.D. Vice-President.—Alexander Marcet, M.D. F.R.S.—James Moore, Esq.—John Pearson, Esq. F.R.S.—P. M. Roget, M.D. F.R.S. Secretary.—A. R. Sutherland, M.D.—H. L. Thomas, Esq. F.R.S. Vice-President.—James Ware, Esq. F.R.S. Vice-President.—Stephen Winthrop, M.D.—John Yelloly, M. D. F. R. S. Vice-President and Treasurer.

*Apothecaries' Bill.*—The *Society of Apothecaries of London,* incorporated by Charter granted in the reign of King James the First, having introduced into Parliament a Bill for enlarging the Charter

Charter of their Society, and for better regulating the practice of Apothecaries throughout England and Wales, beg permission to give a brief statement of their design, and of the motives which have induced them to undertake it.

While the church, and the profession of the law, in all its departments, have been fenced round with such restrictions as are calculated to prevent improper persons entering into them, the medical profession alone has been left entirely unguarded; so that every description of empirics, and of illiterate, uneducated, and uninformed persons, are permitted to undertake the cure of diseases; to the discredit of the profession, the detriment of the regularly-educated practitioner, and, what is of infinitely superior importance, to the incalculable injury of the community, particularly of the lower classes of the people.

These evils have long been the subject of serious consideration; and, under a strong impression of their importance, an Association of the Apothecaries, and Surgeon-Apothecaries of England and Wales, was formed about three years ago, for the purpose of applying to the legislature to obtain a redress of those evils. Under the sanction of this association, a Bill was introduced into parliament in the session of 1813. This Bill was so extensive in its nature, and embraced so great a variety of subordinate objects, that it was considered parliament could not entertain it, and it was therefore withdrawn. Among other objections to this Bill, was that made to it by the Royal College of Physicians, who, though they approved of the general principles upon which the Bill was founded, were of opinion, that the powers contained in it should be vested only in the Society of Apothecaries of London, as established by charter.

This society, convinced of the evils already stated, felt itself imperatively called upon to take them into its serious deliberations; and having carefully examined the charter abovementioned, was of opinion that it contained nearly every thing that was requisite to the removal of these evils, provided its powers were sanctioned by the legislature, extended to the kingdoms of England and Wales, and such few additional powers given as would render it more applicable to the present times.

With this view the present Bill was formed, and the clauses contained in it have been submitted to the Royal College of Physicians, one of which has been introduced at their recommendation.

The principal design of this Bill is to secure to the public, in future, a succession of well-educated and properly-qualified apothecaries, which, when it is considered that the lives and health of more than nine-tenths of the community are intirely under the care of this branch of the medical profession, must be universally allowed to be an object of the highest importance.

The means by which this design is proposed to be carried into effect, is by an apprenticeship for a moderate term of years, succeeded by a suitable attendance on the practice of an hospital or regular regular infirmary, and accompanied by testimonials of a sufficient medical and classical education, and of good moral conduct.

To ascertain the qualifications of the apothecary in these branches of science, a Board of Examiners is to be appointed by the Society of Apothecaries, who are in conjunction with the President of the Royal College of Physicians, or some other fellow or fellows appointed by him, to examine such persons; and, if approved by them, to grant them certificates of qualification, without which no person can in future be permitted to practise as apothecaries.

In this Bill every retrospective effect is particularly guarded against, not only with respect to persons already in practice, but also as it relates to those who have already commenced their medical studies, so that no possible injury can occur to either of them.

It is obvious, therefore, that its beneficial effects will be only gradual, yet there can be no doubt but a very moderate number of years must produce a very decided advantage to all classes of the community, but particularly to those of the middling and inferior ranks, who are at all times much exposed to the arts of ignorant and unprincipled men.

The examination of the shops of apothecaries, to ascertain the quality of their medicines and drugs, has been long practised, in a certain degree, in the metropolis. Another object of this Bill is to extend and facilitate this practice, the great importance of which is too obvious to require illustration.

It may possibly be objected to this Bill, that the necessity under which it puts every person proposing to commence business as an apothecary, to visit the metropolis, for the purpose of examination, must be viewed as an unreasonable hardship; but when it is recollected that London is the grand source of this species of medical education, insomuch that much the greater number of medical students have occasion, upon that account, to come to it from all parts of the kingdom, this objection will be greatly diminished; and, indeed, may be esteemed intirely removed, when it is considered that every attorney in the kingdom is liable to the same inconvenience, being obliged to take a journey to London for a similar purpose of admission to practice.

It may likewise be objected, that this Bill is only a partial remedy for the evils it professes to remove, having no reference to two other important branches of medical practice; namely, surgery and midwifery: but of these the Society of Apothecaries could not take cognizance. The superintendance of the public health, in these particulars, rests with others, with whom it is neither the province nor the wish of the Society of Apothecaries to interfere.

---

The Baron Percy, Member of the Institute, Professor of the School of Medicine at Paris, Inspector-General of the Army, &c. has lately composed a Memoir on the question, whether a Living

Part

Part having been completely separated from the Animal System, is susceptible of being re-united to it?

It seems, that about the middle of the last century, GARENGEOT, surgeon of Paris, a man of character and high reputation, published in a Treatise on Surgery, the following fact: Two soldiers coming out of a tavern, fought in the street; one of them seized with his teeth the nose of his comrade, bit it clean off, threw it in the kennel, and trampled it under his feet. Whilst the wounded man pursued his adversary, the piece of nose was picked up, washed in a fountain, and thrown into the shop of the surgeon Gallain, a friend of Garengeot. When the soldier was brought to him a few seconds afterwards, the detached nose was cold and livid. However, according to Garengeot, after it was washed and warmed in hot wine, and the wound being in like manner cleaned, the nose was returned to its place, and supported by sticking plasters and a bandage, with so much success, that in four days it had firmly united. Notwithstanding the high character of those two surgeons, the account was deemed false and absurd; indeed, it was thought so ridiculous, that Garengeot entirely lost his character. From some other cures of this nature being recorded, the Baron Percy determined to pursue this enquiry. He dwells at some length upon the facts recently stated by Dr. Balfour, and recorded in our Journal, and repeats the ancient stories from Taliacotius, &c. From his own experience, he has proved, that in cases where the nose was divided and hung by a slip of skin, the re-union and cicatrization were easily and completely effected; but when the separation was total, the operation even under the most favourable circumstances, was ineffectual. Messrs. Percy and Richerand attempted to join the extremity of the nose of some dogs which they had carefully cut off; but they did not succeed in the experiment. They were more fortunate with the ears of animals, as in the rabbit, sheep, &c. For this purpose, they envelloped the ear, after it was re-applied, in a paste-board box, and plastered the wound over with pitch of a moderate heat. M. Percy concludes his Memoir, by relating an experiment which he attempted jointly with M. Willaume, Surgeon-in-Chief of the French Army. A portion of the entire diameter of the tibia of a dog, about half an inch long, having been removed, was replaced by a cylinder of platina intended to prevent the shortening of the limb. The whole was envelloped in pitch, and bound up with a proper dressing. At the end of some days, a bony incrustation had begun to form on the cylinder, but the dog escaped, and the experiment was not repeated.—*Gazette de Santé.*

---

The Journal de Medicine for December, gives another account of spontaneous combustion in a defunct drunkard. As such subjects are at least as common in London as in any other metropolis, we shall wait till we meet with a similar combustion before we give our opinion on the subject.

 The

The Medical Society of Marseilles have sanctioned, by their authority, the opinion that meazles may occur a second time in the ame individual.

---

*Case of Inflammatory Catarrhal Head-ach immediately cured by the Moxa applied to the Head;* by M. Bonson, D.M.P.—A Polish servant laboured under catarrhal affection, complicated with icterus, and with a violent pain at the lateral, left, and anterior part of the head, so that he could scarcely remain a few moments in one posture; the pain was so intense, that it was soon considered the principal object of attention.

The patient was stout, young, and strong; he expectorated much mucous. The left eye was closed and watery as in common catarrh; raising the eyelid, *the pupil was found contracted, the conjunctiva inflamed;* the pain was principally in the bottom of the orbit on this side, and towards the temple, and propagated itself to the top of the head. He experienced troublesome pulsations in these parts, and the least pressure was insupportable; the pulse was slow, full, and hard; the surface of the body cold, and especially the hands, whose nails and fingers had acquired a livid tint. He was bled largely, purged and vomited with relief; but the following morning the pain returned. After much ineffectual treatment, I determined on applying moxa to the principal seat of the pain. No sooner had the fire reached the skin than the internal pain was removed; an abundant perspiration diffused itself over the body; the pain appeared no more. The author does not agree with Ponteau and De Haen, in the apprehension of danger from the moxa applied to the head.

---

*Margaret Austin*, widow, having had four children, died in the neighbourhood of Kilkeel, in Mourne, county of Down, on Friday, January 27, 1815, in the 51st year of her age. She had long laboured under a painful and tedious disease with considerable fortitude. Early in the year 1805, she perceived a small tumour in the lower part of the abdomen, sometimes attended with considerable pain, and which continued progressively to increase until the 28th of October, 1812, at which time she underwent an examination before the medical gentlemen of that town. It was then ascertained, that the tumour or excrescence had increased to an amazing size, nearly of a globular form, was moveable, and strongly attached to the uterus, or some of its neighbouring internal parts. A very considerable quantity of extravasated serum was found collected in the abdomen, and the operation of the paracentesis being found necessary, it was performed, by which eighteen quarts of water were then taken off, perfectly inoffensive in its smell, and tolerably clear. The operation was repeated at intervals, when necessary, for fifty-eight times, in the space of two years and three months, during which time the amazing quantity of 1276 quarts, or 312 gallons of water were drawn off. For the the most part of the time she enjoyed tolerable health, eat moderately, slept, and seldom was much troubled with thirst. The day after her death, the body was opened by Messrs. Adderly and Graham, the medical gentlemen who had attended her regularly through the course of the disease. On the tumour being removed from the body, it weighed twenty-pounds and a half, avoirdupois weight; it was nearly of a globular form, somewhat depressed on the top, and perfectly smooth. On the back part, near to its top, it was slightly attached to the mesentery; but at the bottom, from which it first originated, it was strongly joined by broad ligaments and large blood vessels to the uterus, falopian tubes, and the internal extreme part of the vagina; the ovaria appeared to be nearly obliterated. On dissecting the tumour, it appeared to be mostly composed of a hard cartilaginous substance, greatly resisting the knife, and interspersed with innumerable ramifications of small blood vessels, which gave it a most curious appearance. A very considerable portion of the top of the tumour was in a state of putrefaction; and from its long confinement, and in some measure being concocted by the heat of the body, the putrid matter had the appearance of coagulated milk, or the whites of boiled eggs. The uterus, although strongly adhering to the tumour, was of a natural size, and free from disease; all the other viscera of the abdomen appeared sound.

---

We have to lament the death of that celebrated and truly deserving character, James Ware, Esq. of Bridge-street. This gentleman, who has contributed so much to improve the practical part of surgery in his department as an oculist, was not more distinguished as a surgeon than for his enlarged philanthropy. In partnership with the late Mr. Jonathan Wathen, he was probably the first English surgeon who separated this branch of the art from general practice. When Mr Phipps, grandson to Mr. Wathen, became of an age to take part in the practice, the two former separated, Mr. W. uniting with his grandson. It was soon found that the town was large enough for all three. The partners removed to the west, and Mr. Ware continued for the remainder of his life in the city, with a country house at Turnham Green, which enabled him to attend his patients in the west without inconvenience. Apparently of a delicate frame, a premature old age assailed him, though constantly prudent in every part of his life. We shall, in our next, give a more detailed account of his history, but cannot conclude these few remarks without mention of his exemplary liberality to the Society for the Relief of Widows and Orphans, of which he was among the early founders, and for several years president. Besides his customary donation, he presented them during life with a fund, the interest of which was divided among such of the directors as attended the quarterly courts with punctuality, and staid the whole time of necessary business.

We have in one of the newspapers a remark which would lead us to suppose, that Mr. Ware had relinquished the use of laudanum altogether in inflammation of the eye. As his experience increased, it is probable that he varied his remedies; but the *vinous* solution of opium was, we believe, always retained.

---

It is with much concern that we announce the death of Smithson Tennant, Esq. F.R.S. and Professor of Chemistry in the University of Cambridge; a man in whom genius, talents, and virtue, were united in their highest forms. Although his industry was checked by a frame naturally weak, and a languid state of health, his acquirements in science were general, in chemistry most profound, with a correctness not exceeded by Woolaston.

The circumstances of Mr. Tennant's death were most afflicting. He was returning from France, where he had been several months, and was waiting at Boulogne for a favourable wind. He had actually embarked on Wednesday, the 22d of February, but the vessel was obliged to put back; and it was determined, if the weather should be tolerable, to make another trial in the evening. During the interval Mr. T. proposed to a German officer of distinction, whom he had accidentally joined on the road, and who was also going to England, to ride with him to Buonaparte's Pillar, near Boulogne. In returning he deviated a little to look at a fortification near the road. But as they were attempting to pass a draw-bridge, which, owing to some neglect, was not properly secured, the bridge gave way, and they were precipitated into the trench. The officer fortunately escaped without any serious hurt; but Mr. Tennant was found fallen under his horse, and was taken up speechless, his skull and one of his arms being considerably fractured. He was conveyed with difficulty to the hospital at Boulogne, as the nearest and most convenient place to receive him, and expired in half an hour. His remains were interred at Boulogne.

---

The editors feel greatly obliged by communications they have received from two learned correspondents, Dr. Parry, of Bath, and Mr. Blunt, of Birmingham, on the subject of M. M. Wenzel's Corrollaries on Epilepsy. Instead of "Cerebellum," the translator should have written "the pituitary gland." The only apology to be offered is, that during the continental, and afterwards the American war, so many difficulties occurred in procuring foreign Journals, that in order to preserve a faithful register of passing events, it was found necessary to use the assistance of philosophical compilers from the periodical Journals of every description. In consequence of this, the article being selected from an American Journal, was copied and printed verbatim. The original work has been long in London, and was, with much gravity, noticed by a Review now defunct. The editors of the London Medical Journal considered the whole too little deserving notice to make an article, and are happy to find their opinion confirmed by their learned and polite correspondents.

---

We have to return thanks for numerous informations we have

 received, received, concerning the epidemic which has been so general in the flat eastern part of the Island. At Cambridge, it has been most noticed, probably on account of the number of subjects at that celebrated university, who are not natives of that part of England, and also on account of the importance of their lives from the rank they hold in the country. Yet, even here, the number of deaths has not exceeded eight or ten at most. In other parts we do not find the mortality has been so considerable, though the disease has been very general. It has assumed the name of Typhus Mitior or gravior, according to its severity, has no where appeared to be contagious, though attacking many in the same house from the same cause. The students at Cambridge were permitted to leave the university for a term, but for some weeks past have had notice to return, the danger being supposed at an end.

---

Dr. CLUTTERBUCK will begin his Summer Course of Lectures on the Theory and Practice of Physic, Materia Medica, and Chemistry, on Friday, June the 2d, at ten o'clock in the morning, at his house, No. 1, in the Crescent, New Bridge-street, Blackfriars.

The Summer Courses of Lectures on the Theory and Practice of Physic, by Dr. ROGET; and of Materia Medica and Medical Jurisprudence, by Dr. HARRISON; will commence, as usual, in Windmill-street, on the first week in May. The Lectures on Chemistry, will, in consequence of Dr. DAVY's absence from town, be given during the summer by Dr. GRANVILLE.

Dr. SQUIRE will, on Tuesday, the 16th of May, begin a Course of Lectures on the Theory and Practice of Midwifery, and the Diseases of Women and Children.

*Theatre of Anatomy, Bartlett's-court, Holborn.*—The Summer Course of Lectures on Anatomy, Physiology, Pathology, and Surgery, by Mr. JOHN TAUNTON, F. A. S. Member of the Royal College of Surgeons of London, Surgeon to the City and Finsbury Dispensaries, City of London Truss Society, &c. will commence on Saturday, May 20th, 1815, at eight o'clock in the evening precisely, and be continued every Tuesday, Thursday, and Saturday, at the same hour.

Theatre of Anatomy, Great Windmill-street.—Mr. SHAW will commence a Course of Anatomical Demonstrations, towards the end of May.

---

The LONDON PRICES of DRUGS were, on the 21st of April, as given in our last, excepting the following articles:

| | £. | s. | d. | | £. | s. | d. | | £. | s. | d. | | £. | s. | d. |
|---|---|---|---|---|---|---|---|---|---|---|---|---|---|---|---|
| Cantharides.... | 0 | 10 | 6 | to | 0 | 11 | 0 | Saffron, Spanish | 2 | 10 | 0 | to | 2 | 14 | 0 |
| Cream of Tartar | 5 | 15 | 0 | | 6 | 6 | 0 | Scammony, Al. | 1 | 10 | 0 | | 1 | 11 | 0 |
| Tragacanth.... | 27 | 0 | 0 | | 27 | 10 | 0 | Verdigris, Dry . | 0 | 6 | 6 | | 0 | 6 | 9 |

*Price of Vials per Gross.*—8 oz. 70s.—6 oz. 58s.—4 oz. 47s.—3 oz. 43s.—2 oz. 36s.—1 oz. 30s.— ½oz. 24s.

Lisbon Leeches, 30s. per hundred.—True French ditto, 55s.

Essential Salt of Lemons, 4s. 6d. per dozen.

METEORO-

### METEOROLOGICAL REGISTER.

### *From March the 23d, to April the 25th,* 1815.

Kept by C. BLUNT, Philosophical Instrument Maker, No. 38, Tavistock-Street, Covent-Garden.

| Moon. | Day. | Wind. | Barometrical Pressure. | | | Temperature. | | | |
|---|---|---|---|---|---|---|---|---|---|
| | | | Max. | Min. | Mean. | Max. | Min. | Mean. | |
| | 26 | W | 29·45 | 29 45 | 29·45 | 57 | 47 | 52 | Rain |
| | 27 | SW | 29 50 | 29·44 | 29·466 | 58 | 48 | 53 | Rain |
| | 28 | S | 29 90 | 29 57 | 29·772 | 58 | 49 | 53·5 | Fair |
| | 29 | S | 29 97 | 29 85 | 29 892 | 60 | 50 | 55 | Fair |
| | 30 | S | 29·97 | 29 91 | 29·935 | 61 | 52 | 56·5 | Fair |
| | 31 | W | 29·92 | 29 82 | 29 865 | 62 | 51 | 56·5 | Fair |
| ◑ | 1 | SW | 29 67 | 29·63 | 29·646 | 63 | 52 | 57·5 | Fair |
| | 2 | S | 29·8 | 29·8 | 29 8 | 64 | 55 | 59 5 | Fair |
| | 3 | S | 29 89 | 29 83 | 29·847 | 65 | 55 | 63 | Fair |
| | 4 | S | 29 96 | 29 91 | 29·93 | 65 | 56 | 60·5 | Fair |
| | 5 | SE | 30 22 | 30·18 | 30 202 | 65 | 54 | 59·5 | Fair |
| | 6 | SE | 30·19 | 30 11 | 30 152 | 63 | 54 | 58·5 | Fair |
| | 7 | E | 30·05 | 29·99 | 30·017 | 63 | 52 | 57·5 | Fair |
| | 8 | E | 29·94 | 29·87 | 29 89 | 61 | 50 | 55·5 | Rain |
| ● | 9 | E | 29 88 | 29 88 | 29·88 | 61 | 50 | 55 5 | Fair |
| | 10 | E by N | 29·89 | 29 85 | 29·872 | 60 | 48 | 54 | Rain |
| | 11 | E | 29 96 | 29·96 | 29 96 | 58 | 45 | 51·5 | Fair |
| | 12 | NE | 29·93 | 29·85 | 29·832 | 56 | 39 | 47·5 | Fair |
| | 13 | SE | 29·75 | 29·60 | 29 645 | 55 | 37 | 46 | Thurder. Hail, & Rain |
| | 14 | SW | 29 83 | 29 58 | 29·702 | 52 | 35 | 43·5 | Rain & Sleet |
| | 15 | N | 30 08 | 30 04 | 30·055 | 53 | 35 | 44 | Snow |
| ◐ | 16 | N | 30 10 | 30·10 | 30 10 | 53 | 34 | 43 5 | Rain |
| | 17 | N | 30 1[illegible] | 30·12 | 30 142 | 53 | 32 | 42·5 | Rain |
| | 18 | N | 30·25 | 30·22 | 30·232 | 53 | 34 | 43·5 | Rain |
| | 19 | N | 30·16 | 29 95 | 30·06 | 54 | 36 | 45 | Rain |
| | 20 | N | 29 83 | 29 7 | 29·762 | 55 | 37 | 46 | Rain |
| | 21 | N | 29 25 | 29 87 | 29·02 | 55 | 33 | 44 | Rain |
| | 22 | N | 29.09 | 28 88 | 28 982 | 54 | 34 | 44 | Rain |
| ○ | 23 | N | 29 20 | 29·20 | 29·20 | 54 | 34 | 44 | Rain |
| | 24 | N | 29·52 | 29·33 | 29·415 | 55 | 33 | 44 | Rain |
| | 25 | N | 29·69 | 29 67 | 29 68 | 55 | 33 | 44 | Fair |

RESULTS.

| Mean barometrical pressure of the month | | 29·772 | Mean temperature of the month | | 48·556 deg. |
|---|---|---|---|---|---|
| Maximum 30·25 | wind at | N | Maximum 65, | wind at | S |
| Minimum 28·88 | ——— | N | Minimum 32, | ——— | N |

*Scale exhibiting the prevailing Winds during the Month.*

| N | NE | E | SE | S | SW | W | NW |
|---|---|---|---|---|---|---|---|
| 11 | 1 | 5 | 3 | 6 | 3 | 2 | 0 |

| | Mean barometrical pressure. | Mean temperature. |
|---|---|---|
| From the full moon on the 25th March, to the last quarter on the 1st April | 29·718 | 54·85 |
| ——— last quarter on the 1st, to the new moon on the 9th | 29 964 | 58·75 |
| ——— new moon on the 9th, to the first quarter on the 16th | 29·88 | 47·14 |
| ——— first quarter on the 16th, to the new moon on the 23d | 29·771 | 44·14 |

REPORT

### REPORT OF DISEASES.

SINCE the alteration in the weather, which will be apparent by consulting our Meteorological Register, Catarrhal and Pulmonary Affections have increased. Sore-throat, not of a malignant character, has occurred frequently. Several cases of Erysipelas, in some of which the head and face were swelled, in one instance so much so as to occasion blindness, have come under the Reporter's immediate notice; and Rheumatism, chiefly in the chronic form, has lately claimed his frequent attention. In this disease, it is often easier to say what remedies have not been serviceable, than to enumerate those which have produced benefit. Bark, either alone or conjoined with guaiacum, so much extolled by many writers, has little efficacy in the rheumatic affections, which, during the cold months, are continually occurring in public practice. A combination of antimony, calomel, and opium, seems to succeed the best; the bowels being kept open with suitable purgatives, and stimulating liniments applied to the affected parts, and afterwards bark, with the view of restoring the debility in which the patient is left when the more painful symptoms have abated.

A case of chorea, still under care, is attended with some peculiar phenomena. The patient, a girl aged nine years, of delicate habit, when two years old, became unable to walk like other children of her age, and her legs were observed to bend. She recovered from this weakness, however, under the care of a surgeon, and continued very well till last November, when she began to complain of great pain in the back part of the neck. This increased, and she became affected with an inability to move her limbs; and, six weeks ago, was seized with the startings and violent motions of St. Vitus's Dance. Such was the short history related by the child's parents. Upon examining the little sufferer, the left side was evidently paralytic, she dragged the leg of that side along with difficulty, the limb making semicircular movements, and then advancing with a sort of jumping stride of unequal length, and out of the child's power to command. The symptoms of Chorea were betrayed by the tossing and unmeaning motion of the arms, twisting of the trunk and right leg. The head stooped forward and downwards towards the sternum from the neck; the pupils of the eyes were much dilated, the face of a florid hue, and a slight degree of hesitation of speech was slightly perceptible; the child was duller and more stupid than girls of her age commonly are. The bowels were constipated; the belly hard, but not enlarged; and the stools were of a dark colour. The last cervical vertebra evidently projected; and an increase of pain was complained of when pressure was made upon it.

The treatment at present insisted upon is simply a purge of calomel and rhubarb each other morning, and constantly blistering the neck. This has been continued about a fortnight, and has already been productive of benefit. The fæces present a more natural appearance, and the child possesses a little more power over her limbs. In a future Report, the history of the case will be continued.

